# Predicting Protein Kinase Specificity: Predikin Update and Performance in the DREAM4 Challenge

**DOI:** 10.1371/journal.pone.0021169

**Published:** 2011-07-28

**Authors:** Jonathan J. Ellis, Boštjan Kobe

**Affiliations:** School of Chemistry and Molecular Biosciences, Institute for Molecular Bioscience and Centre for Infectious Disease Research, University of Queensland, Brisbane, Queensland, Australia; John Innes Centre, United Kingdom

## Abstract

Predikin is a system for making predictions about protein kinase specificity. It was declared the “best performer” in the protein kinase section of the Peptide Recognition Domain specificity prediction category of the recent DREAM4 challenge (an independent test using unpublished data). In this article we discuss some recent improvements to the Predikin web server — including a more streamlined approach to substrate-to-kinase predictions and whole-proteome predictions — and give an analysis of Predikin's performance in the DREAM4 challenge. We also evaluate these improvements using a data set of yeast kinases that have been experimentally characterised, and we discuss the usefulness of Frobenius distance in assessing the predictive power of position weight matrices.

## Introduction

Linear motifs — short, functional regions of proteins — play a vital role in signalling and the regulation of cellular processes [1], [2]. Many different classes of linear motifs have been identified and catalogued [3]. One of the best characterised classes of linear motifs are phosphorylation sites. Phosphorylation — the transfer of a phosphate group from a phosphate donor onto an acceptor amino acid – is a ubiquitous regulation event that acts as a switch turning proteins “on” or “off” and propagating signals through the cell. Phosphorylation of proteins is controled by protein kinases, a large super-family of proteins [4]–[6]. Several families are shared across many of the eukaryotic phyla, and it has been possible to trace the evolutionary path of these families [7]. The human genome contains 518 predicted protein kinases [8], and it is estimated that up to 30 percent of the human proteome may be phosphorylated at some point [9]. Hundreds of these kinases have been linked to cancers [10]; this has made protein kinases intensively studied drug targets [11].

Experimental determination of kinase specificity is both expensive and time-consuming, and identification and validation of substrates can be even more laborious [12]. This is partly due to the transient nature of the interaction — a necessary attribute of an efficient regulatory network — making it difficult to determine the kinase responsible after the fact. Substrate identification still remains one of the rate-limiting steps in understanding the function of novel protein kinases.

Traditional computational domain recognition techniques are not well suited for identification of phosphorylation sites, and linear motifs in general, due to their short nature — typically less than 12 residues — and the probability of seeing false positives is always very high. Furthermore, the specificity of a protein kinase is determined not only by peptide specificity — the phosphorylation residue preference and composition of surrounding residues [12] — but also by the substrate recruitment mechanisms and, more generally, the context that the kinase finds itself in, and substrate recruitment [13].

We have previously described an algorithm, Predikin, for predicting peptide specificity of protein kinases and identifying substrates for protein kinases based on the concept of specificity-determining residues (SDRs) [14]–[16] (see Methods). In this article, we present further enhancements to the prediction algorithm, and evaluate them against a set of protein kinases from *Saccharomyces cerevisiae* [17]. We also report on the success of Predikin in the recent DREAM4 (Dialog for Reverse Engineering Assessments and Methods) community challenge[18]. In this challenge entrants were asked to predict the specificity of previously uncharacterised protein kinases, and the predictions were compared to experimental data. For all kinases that made up the challenge, Predikin produced the most accurate predictions.

## Results

**Improvements to Predikin**. We have recently made some improvements to the Predikin algorithm and website. These include the ability to select different substitution matrices, the streamlining of the website to allow easier prediction of potential kinases given a substrate and the ability to perform whole-proteome analysis.

**Substrate-to-Kinases Predictions**. There are two fundamental questions a researcher may wish to ask about phosphorylation: which proteins will be phosphorylated by kinase X? and which kinases will phosphorylate protein Y? This is essentially the same problem, but seen from two different directions, and Predikin is able to answer both. Predikin's approach is the same regardless: analyse the kinase to produce a position weight matrix and then use this to score a potential phosphorylation site. However, in previous versions of the web server submitting one substrate and many kinases was not very practical. The web server has been redesigned so that now a researcher only needs to submit a single sequence file, the content of which determines the type of analysis: the file may contain one kinase and multiple substrate to identify likely targets of the kinase, or it may contain multiple kinases and one substrate to identify the most likely kinase. Multiple kinases with multiple substrates may also be submitted for larger analysis. Predikin attempts to align each sequence to a hidden Markov model describing the kinase catalytic domain. This information is used to identify which sequences in the submitted file are kinases, and thus a researcher need not specifically identify which are kinases and which substrates. All submitted sequences are treated as potential substrates, and thus auto-phosphorylation, or phosphorylation by another kinase can also be detected.

**Whole-Proteome Analysis**. The Predikin web server has also been adapted to allow large scale analysis to be conducted. This makes it possible to scan whole proteomes rather than a subset of selected proteins. As Predikin identifies kinase sequences, it is possible to submit a whole proteome in FASTA format and allow Predikin to identify the kinases and score each one against every potential phosphorylation site in the proteome. This type of analysis can be very time consuming (depending on the number of sequences, number of kinases and number of potential phosphorylation sites); therefore, these jobs are queued and the results emailed to the researcher. Smaller jobs are still run on-demand and the results are presented to the researcher through the web site. In the future, we intend to make the results of whole proteome analysis available on the Predikin website. This will enable researchers to access the results of common queries much faster.

**Extending Predikin's Reach**. Prior to submitting predictions to DREAM4, Predikin was unable to build valid weight matrices for two of the three protein kinases in the DREAM4 challenge. This led to three changes to the system that all contribute to increasing the number of protein kinases Predikin can build position weight matrices for: Updating PredikinDB, changing substitution matrices and changing substitution matrix cut-off values.

PredikinDB has continued to be updated from the latest UniProtKB [19] releases. It now also incorporates data from PhosphoELM [20]. Including further data sources has significantly increased the number of protein kinase-substrate interactions in PredikinDB — it now contains 5127 phosphorylation sites that are linked to a specific kinase, 2260 from PhosphoELM and 2867 from UniProtKB — this increases the chances of building a valid frequency matrix (see Methods); therefore, Predikin is now able to make predictions for a much broader range of protein kinases.

To assess the ability of these new features to increase the number of protein kinases Predikin can make predictions for, and to evaluate their affect on accuracy, a published data set of 61 protein kinase from yeast was used. For each of these kinases, a position weight matrix, which described the sequence specificity surrounding the phospho-residue, had been experimentally determined [17].

To successfully build a position weight matrix, the Predikin method relies on identifying similar specificity-determining residues, and this, in turn, is reliant on the substitution matrix used. Testing has shown that the use of different substitution matrices can enable Predikin to build position weight matrices for more protein kinases (by altering what Predikin considers similar to a specificity-determining residue). To analyse the benefits of using different substitution matrices, we attempted to build position weight matrices for each of the yeast protein kinases using various BLOSUM matrices. To assess the quality of Predikin's position weight matrices we used the same evaluation method as the DREAM4 challenge: similarity to a experimentally mapped position weight matrix using the distance induced by the Frobenius norm (Frobenius distance; see Methods). The DREAM4 challenge also provided p-values for each Frobenius distance, this is the probability that a random position weight matrix has the same or smaller Frobenius distance, and we have applied the same method to calculate p-values for the yeast kinase predictions.

**Figure 1:**
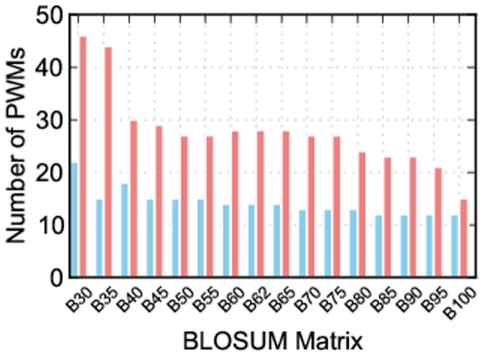
Number of position weight matrices built using each BLOSUM matrix. Each bar represents the number of kinases for which a position weight matrix could be built using each of 16 BLOSUM matrices. The blue bars show the number of position weight matrices built when using a cut-off value of 1, and the red bars show the number when using a cut-off value of 0. When considering just the number of position weight matrices, BLOSUM30 is clearly superior, and this is even more apparent when using a cut-off value of 0.

From 16 BLOSUM matrices, BLOSUM30 clearly stands out as providing the most position weight matrices (Figure 1), but an important question is whether the position weight matrices produced by this matrix are as accurate as those built by Predikin's default substitution matrix: BLOSUM62?

**Figure 2:**
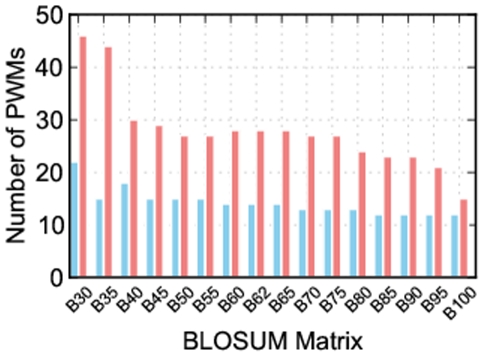
Using different BLOSUM matrices does not adversely effect Frobenius distance. The Frobenius distances achieved for 12 yeast kinases with various BLOSUM matrices using a cut-off value of 1 are shown. Each line represents one kinase; altering the BLOSUM matrix does not have a significant effect on distance as can be seen by the predominately horizontal lines.

We calculated the Frobenius distance for the 12 protein kinases for which a position weight matrix can be built using all of the substitution matrices. For any given kinase, the distance produced does not vary greatly as the BLOSUM matrix changes (Figure 2). These results also show that there is no single best substitution matrix – the best matrix to use is dependant on the kinase (and there is no way of knowing in advance which matrix will perform best), and that while we may not select the best matrix for individual kinases every time, the difference in the prediction is likely to be very small.

Together these results show that we are able to increase the number of kinases Predikin can build position weight matrices for by changing the substitution matrix, and that BLOSUM30 captures the most kinases. We have also shown that the distance to the experimentally derived position weight matrix is not adversely effected by the use of BLOSUM30. We have also found that altering the substitution matrix cut-off value affects the number of position weight matrices that can be built. BLOSUM62 contains numbers ranging from −4 to 11 with higher numbers indicating more likely substitutions; by default, Predikin uses a cut-off value of 1, meaning that any substitution with a positive score is allowed; however, using a cut-off value of 0 greatly increases the number of kinases that position weight matrices can be built for, without affecting the accuracy of those position weight matrices. By using a cut-off value of 0 Predikin is able to build position weight matrices for many more protein kinases (Figure 1).

**Figure 3:**
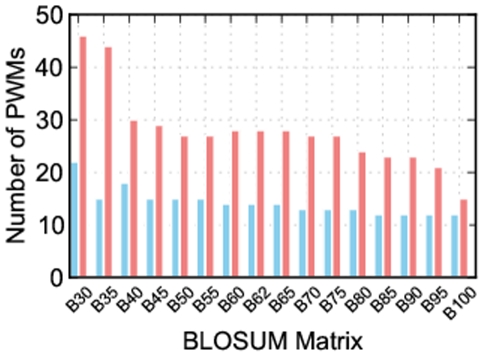
Using a BLOSUM cut-off value of 0 instead of 1 does not adversely effect Frobenius distance. The Frobenius distance is shown for 12 kinases using BLOSUM62 and a cut-off value of 1 (blue) and 0 (red). In each case it is apparent that switching from a cut-off value of 1 to 0 has little effect on the Frobenius distance.

We also asked the question of whether using a cut-off value of 0 adversely affected the distances we obtained compared with using a value of 1. We calculated the distance from the experimentally derived position weight matrix for 12 kinases using a cut-off value of both 1 and 0. In four cases, the smallest distance was produced with a cut-off value of 1 (Cdc5, Gcn2, Hrr25 and Ste20) and, in a further four cases, a cut-off value of 0 gave the smallest distance (Tpk1, Tpk2, Tpk3 and Ypk1). In the remaining four cases (Cla4, Ipl1, Pkh2 and Prk1) the smallest distance was equal between cut-off values (Figure 3). These results show that using a substitution cut-off value of 0 does not adversely affect the majority of cases — and in some cases it even improves the Frobenius distance obtained. Again, the advantages of extending the range of Predikin are significant, while the disadvantages in increases to distance are very slight, as in most cases the increase in distance is itself very small.

**Figure 4:**
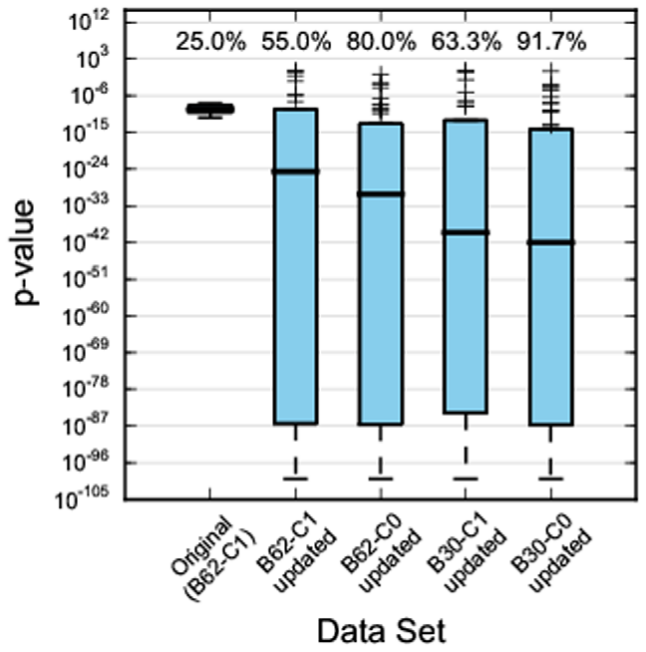
Effect of various BLOSUM matrix and cut-off values on Predikin's performance. Each boxplot shows the distribution of p-values obtained from the set of 61 yeast protein kinase from [17]. The left-most, original, plot shows scores obtain with the version of Predikin that were used for the DREAM4 predictions. The next plot shows the distribution when the new method of DREAM4 position weight matrix is applied to the original database, and the following plots show the distributions obtained with the updated database (including PhosphoELM data) using different Predikin options. B62/B30 indicates BLOSUM62 and BLOSUM30 as substitution matrices, respectively, and C1/C0 indicates a BLOSUM cut-off score of 1 or 0, respectively. The numbers above each boxplot show the coverage of each distribution, that is the percentage of the kinases that Predikin was able to make predictions for. The median p-value clearly decreases moving from left to right indicating a general improvement in prediction accuracy. But strikingly, there is a dramatic improvement in coverage – the original algorithm only had a coverage of 25% whereas the right-most distribution (using BLOSUM30 and a cut-off of 0) has a coverage of over 90%.

Figure 4 shows the effect of applying various new options of Predikin to the yeast kinases characterised by Mok et al. [17]. The leftmost distribution, showing output from the original version of Predikin, shows that while all predictions made had good p-values (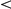
1e-6) Predikin was only able to make predictions for 25% of the kinases. By updating PredikinDB, but still using BLOSUM62 and a cut-off value of 1, Predikin is able to more than double the number of kinases predictions can be made for. The updated database also causes the median p-value to drop quite significantly (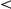
1e-24). This trend is repeated when we use BLOSUM62 with a cut-off value of 0: the median p-value drops below 1e-30 and the coverage of kinase that Predikin can make predictions for rises to 80%. When we switch to BLOSUM30 we see a similar effect, with the final distribution in Figure 4 (far right) showing results using BLOSUM30 and a cut-off value of 0. Here the median p-value drops to 1e-42 and the coverage reaches over 90%. When we use the updated version of PredikinDB, the predictions generally improve, but we also see some outliers starting to appear. These always correspond to kinases that Predikin was previously unable to make predictions for. We consider the benefits of smaller Frobenius distances for most kinases and significantly greater coverage of kinases to greatly out-weigh the disadvantages of a small number of larger distances.

There remained five kinases that Predikin was unable to build specificity matrices for under any circumstances: Cak1, Kin1, Psk1, Sky1 and Ypl141c. Two of these (Cak1 and Sky1) are CMGC (a family of kinases including cyclin-dependent kinases, mitogen-activated kinases, CDK-like kinases and glycogen synthase kinases) kinases and the others are calmodulin-dependent kinases (CaMK). These are the two most represented groups in the kinases (37% CaMK and 25% CMGC kinases), and there are no consistent patterns with the specificity-determining residues of the kinases; therefore, we believe that the inability of Predikin to make predictions for these kinases is simply due to a lack of kinases with similar specificity-determining residues in PredikinDB, and that this will be rectified in time as our knowledge of kinase-substrate interactions grows.

### New Style Position Weight Matrices

**Figure 5:**
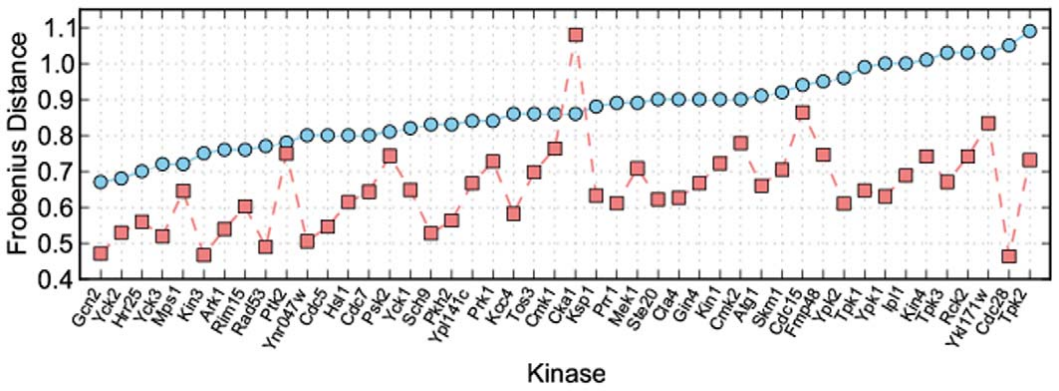
Comparison of old- and new-style position weight matrices. The blue circles show the Frobenius distance for yeast protein kinases achieved using the old style Predikin position weight matrices sorted into ascending order. The red squares show the corresponding distance using the new style position weight matrix. In all cases except one the new style position weight matrix produces a smaller distance than the old style as demonstrated by the green line being below the red.

During the course of our investigations, a different method of converting a frequency matrix to a position weight matrix was devised (see Methods). Figure 5 shows the Frobenius distances for the yeast protein kinases used above where the position weight matrices have been built with both the old (submitted to the DREAM4 challenge) and new methods. For all kinases except one — Cak1 — there is a decrease in distance. We believe that the reason for this improvement is that there were no adjustments for the background amino acid frequencies made with the experimental data; therefore, by also not accounting for them, our predictions more closely mimic the experimental results (see Methods).

**Figure 6:**
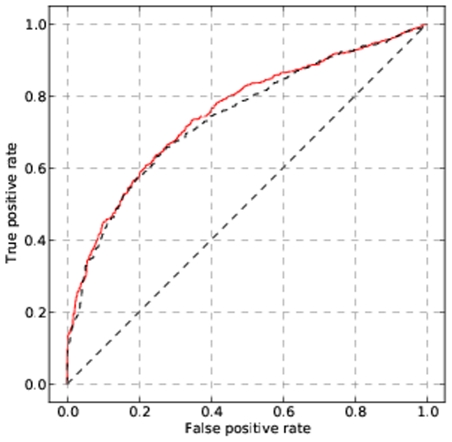
Predictive performance of old- and new-style position weight matrices. The predictive power, as assessed by the area under the ROC curve analysis, of the new-style matrices (black dashed) is virtually identical to that of the old-style (red solid). Demonstrating that Frobenius distance does not necessary provide an insight as to which weight matrix is the best for predictive purposes.

The newer style matrices show a general trend to lower Frobenius distances, and hence lower p-values. As the primary purpose of Predikin is to enable predictions of phosphorylation events, we investigated whether this decrease in Frobenius distance correlates with an increase in predictive power. ROC analysis comparing the two styles of position weight matrix shows that there is almost no difference in predictive power between the two styles of position weight matrix (Figure 6). This results demonstrates that while the Frobenius distance may be useful in determining which of several predicted position weight matrices is closest to an experimentally determined position weight matrix, it does not necessary correlate well with the predictive power of those position weight matrices.

We further investigated the usefulness of the Frobenius distance and associated p-values by testing artificial position weight matrices that show no sequence preference against the protein kinases from the DREAM4 challenge. We constructed three position weight matrices had equal probabilities for all amino acids in all positions (values of 0.05 represent equal probability between the 20 amino acids) except for the phospho-residue position. One weight matrix had probabilities of 0.05 for all amino acids, the second had probabilities of 0.5 for serine and threonine and 0 for all other amino acids in the phosphorylated position, and the third had probabilities of 0.33 for serine, threonine and tyrosine and 0 for all other amino acids in the phosphorylated position. The lowest Frobenius distances was obtained by only assuming the phospho-residue is either serine or threonine — the p-values for these matrices are all lower than the ones obtained by Predikin in the DREAM4 challenge (Table 1).

**Table 1:** Frobenius distances and p-values for low specificity position weight matrices.

	M1		M2		M3	
Kinase	Distance	p-value	Distance	p-value	Distance	p-value
MELK	0.9492	2.12e-3	0.6716	1.33e-28	0.7859	2.44e-15
BIKE	0.9817	1.75e-3	0.7167	6.64e-39	0.8249	5.39e-19
CAMKK2	0.9765	1.15e-3	0.7096	7.48e-25	0.8187	1.19e-14

M1 is a position weight matrix with 0.05 probability for all amino acids in all positions; M2 is a matrix with 0.05 probability for all amino acids in all positions except the phosphorylated residue where P(S) = 0.5 and P(T) = 0.5 and M3 is a matrix with 0.05 probability for all amino in all position except the phosphorylated residue where P(S) = 0.33, P(T) = 0.33 and P(Y) = 0.33.

**Figure 7:**
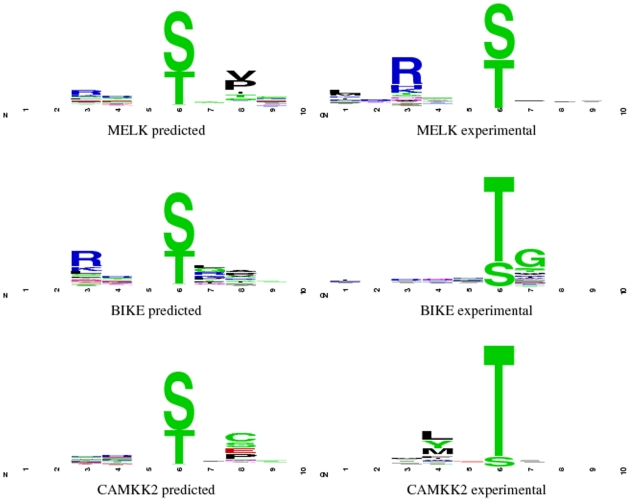
Sequence logos based on predicted and experimental position weight matrices for the kinases in the DREAM4 challenge. The height of symbols within each stack reflects the kinases relative preference of the corresponding amino acid at that position. (Logos were produced with WebLogo [24].)

It is important to remember that some protein kinases are less specific than others, and that in situations involving these kinases a position weight matrix where many of the probabilities are close to 0.05 may be entirely appropriate. To see if this was the case for the kinases in the DREAM4 challenge we produced sequence logos [21] based on the predicted and experimental position weight matrices (Figure 7). All of the kinases in the DREAM4 challenge have positions either side of the phospho-residue that do not have significant amino acid preferences, and that, apart from the phospho-residue position, only one or two other positions have a significant effect on specificity.

### Predikin's Performance in DREAM4

The Predikin algorithm entered the recent DREAM4 challenge and was declared “best performer” in the protein kinase section of the Peptide Recognition Domain specificity prediction category. In the following discussion, it should be noted that the DREAM4 predictions were made before some of the new features of Predikin described above had been implemented and before the evaluations with the yeast kinases had been completed. We were, therefore, unable to take full advantage of the knowledge subsequently gained.

There were three protein kinases in the Peptide Recognition Domain specificity section of the challenge: MELK , BIKE and CaMKK2. In all three cases, the Frobenius distance produced from Predikin's position weight matrix was the lowest achieved by any of the challenge entrants. By default, Predikin used BLOSUM62 as its substitution matrix with a cut-off value of 1. For some of the kinases in the DREAM4 challenge we had to adjust these settings. We used the following: BLOSUM62 with a cut-off value of 1 for CaMKK2, BLOSUM62 with a cut-off value of 0 for MELK and BLOSUM35 with a cut-off value of 0 for BIKE. Table 2 shows Predikin's results from the DREAM4 evaluation; the p-values associated with each distance show that Predikin is producing position weight matrices that are significantly closer to the experimental position weight matrices than would be expected by random. Table 2 also compares the distances achieved with the new form of position weight matrix described above with the distances from the position weight matrices submitted to the DREAM4 challenge. There is considerable improvement for two of the three, but there is a small increase in distance for CaMKK2. This increase for CaMKK2 is because the original position weight matrix did not distinguish between serine and threonine and gives them equal weight; however, the new position weight matrix incorrectly weights serine higher than threonine. The experimental position weight matrix for CaMKK2 shows that it has a very strong preference for threonine as the phosphorylated residue. The new predicted position weight matrix shows serine being more strongly preferred. This error in identifying the phosphoresidue preference accounts for the slight increase in distance for the new predicted position weight matrix compared to the original.

**Table 2:** Frobenius distances for Predikin position weight matrices built with the submitted and new method.

	Submitted Method		New Method	
Kinase	Distance	p-value	Distance	p-value
MELK	0.869	4.181e-08	0.694	9.541e-26
BIKE	0.913	2.055e-08	0.854	5.844e-15
CaMKK2	0.916	3.457e-07	0.938	7.536e-07

The table shows Frobenius distances for position weight matrices built with the submitted and new method. In two of three cases there is a very significant improvement in p-value, while in the third case there is a very small increase in distance.

## Discussion

Predikin was the best performer in protein kinase section of the Peptide Recognition Domain category of the recent DREAM4 challenge: meaning that is was able to predict the experimentally obtained position weight matrix more accurately than any other entrant. This was true for each kinase that comprised the challenge.

Visualisation of the weight matrices, through sequence logos, reveals that there is a mixture of cases where Predikin predicts the specificity reasonably well and cases where there is still room for improvement. Even though Predikin sometimes fails to predict the correct specificity, there are no superior predictors currently available, especially when the repertoire of kinases it can make predictions for is considered. Existing predictors with better reported performance than Predikin have a more restricted repertoire of protein kinase for which they can make predictions, generally because they can only make predictions for kinases with available experimental information on their specificity. Predikin is much less restricted in this regard, it does not require any prior knowledge about the kinases specificity. This makes Predikin an invaluable resource when the protein kinase under consideration is not one of those that has been previously characterised. It should also be noted that there is more to recognition than solely binding of a specific sequence motif to the kinase (i.e., peptide specificity) alone [12]; recruitment also plays an important role. Recruitment can be described as any process that brings a kinase and substrate together, for example, through co-expression and co-localisation. Therefore, a purely sequence based approach will never be able to fully model protein kinase networks, and systems that combine all of these features need to be developed.

The three reported improvements to extend the repertoire of protein kinases Predikin can handle were successful in increasing the number of kinase from the yeast data set from 25% to over 91%, and we have shown that while these changes do not increase the prediction accuracy, of the system they do not adversely affect it either. We developed a method of producing weight matrices that gave lower Frobenius distances, and much lower p-values, than our original method. However, testing revealed that the drop in Frobenius distance did not correspond to an increase in prediction accuracy, as assessed by the area under the ROC curve. One reason for this discrepancy is that one only needs to correctly (or near correctly) predict amino acid specificity for one site but not others to obtain a result that would score as significantly different from random. We also showed that by using a weight matrix that showed no sequence preferences we could obtain very low p-values, but on the other hand such a matrix contains no information about specificity.

From sequence logos derived from the experimentally determined weight matrices it can be observed that usually a kinase only has a well-defined specificity at one or two residue positions. This means that many small changes to other positions (to bring them closer to 0.05 for all amino acids) may have a big effect on Frobenius distance, but provide little useful information regarding specificity.

While Frobenius distance and p-value may be useful in determining which of several matrices is closest to the experimental one, they do not provide a good indication of predictive power or indicate the likelihood of the matrix representing the true position weight matrix. The Frobenius distance suffers from the same problem as other statistics that reduce data to a single global measure in that it does not give local information i.e., there may be local areas that are accurate but some that are not. Ultimately the best measure of accuracy depends on what the weight matrix is intended to be used for. In the case of Predikin it is to make predictions about potential phosphorylated substrates; therefore, the best measure of success is the ability of the weight matrices to identify true phosphorylation substrates. However, this requires a different type of experimental evidence with which to test the matrices – data about which kinases phosphorylate which substrates, rather than an experimentally determined weight matrix, and this is often not available.

### Conclusion

Predikin continues to improve and is a valuable resource for researchers working with protein kinases. Predikin has outperformed other kinase specificity prediction algorithms in an independent test of unpublished data. This combined with several major improvements to the Predikin web server — easier substrate-to-kinase predictions, proteome analysis and new techniques to increase the number of kinases Predikin can work with — make Predikin an important part of a kinase researchers toolbox. The performance of some of the new features has been evaluated against previously published data on yeast protein kinases. We find that these improvements dramatically increase the number of kinase that Predikin is able to make predictions for, and that the accuracy of those predictions is not adversely affected. However, we also find that the evaluation method used in DREAM4 is not necessarily the most appropriate to identify the best predictors.

## Methods

### Predikin's Approach to Kinase Specificity Prediction

Predikin predicts peptide specificity of protein kinases by building a position weight matrix and then using this matrix to score potential phosphorylation sites. For Predikin, a position weight matrix is a 20×7 matrix where each column represents one residue position in a potential substrate with the phosphorylated residue position represented by column 4 (that is, Predikin considers the −3 to +3 residue positions relative to the phosphorylated residue). Each row of the position weight matrix represents one of the twenty amino acids. Individual weights represent the likelihood of a particular amino acid occurring at the specific position in a phosphorylated substrate.

The core of Predikin's approach is the concept of specificity-determining residues. A specificity-determining residue is a conserved amino acid residue, located in the catalytic domain of a protein kinase, that determines what substrate residues will be preferred at a particular position. When a kinase binds to a substrate, the substrate amino acid residues at positions −3 to +3 relative to the phosphorylated residue make contact with specificity-determining residues in a binding pocket on the surface of the kinase. The nature of the specificity-determining residues determines which residues are most likely to be found around the phosphorylation site — that is, which residues “fit” best in the binding pocket. The binding pocket, therefore, makes a major contribution to the specificity of the kinase for different substrates.

Specificity-determining residues where chosen on the basis of an analysis of the crystal structures of peptide complexes of protein kinases, and the location of key binding residues were defined in relation to structural features and conserved sequence motifs [14]. During this analysis we observed a different in the specificity-determining residues of the CMGC group of kinases that warranted their inclusion in a separate class.

The input to Predikin is a protein sequence in FASTA format. Predikin attempts to identify a kinase catalytic domain in the sequence by matching it to the SMART [22], [23] serine/threonine protein kinase hidden Markov model (SM00220) and to one of three patterns to identify the type of kinase (which may be either serine/threonine, CMGC or tyrosine). Alignment to the hidden Markov model is an essential part of identifying the specificity-determining residues for the kinase, if Predikin fails to align the sequence then it will fail regardless of whether the sequence is a true protein kinase. Predikin then searches, in a purpose built database (PredikinDB[15]), for protein kinases with similar specificity-determining residues to the query kinase and builds a frequency table of the number of times each amino acid appears at each of the −3 to +3 positions. Whether a kinase has similar specificity-determining residues to the query kinase is determined using a substitution matrix (by default BLOSUM62). Finally, the frequency table is converted into a position weight matrix by
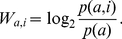
(1)


The background frequency of residue 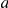, 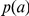, is estimated as its frequency in all substrate sequences in the Predikin database for each kinases type. The frequency of a residue at position 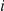 in the substrate, 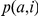, is estimated using pseudo-counts by adding 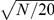 to the raw frequency 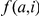 and dividing by 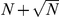 (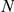 is the number of sequences used to calculate the frequency).

PredikinDB is constructed from data extracted from the UniProt and phospho.ELM databases; although, it can only extract data when a specific kinase is linked to a phosphorylated residue, and in many cases this level of information is not available. It stores information about phosphorylation events and links these to specific protein kinases. Information about the specificity-determining residues for each kinase is also contained in the database. PredikinDB is regularly updated in an automated fashion, and constitutes an important phosphorylation data resource in itself.

### Old and New Style Position Weight Matrices

The old-style weight matrices were created by normalising the matrices produced by Predikin described above so that for each position the weights summed to a total probability of 1. The new method calculates the frequency of each amino acid in the same way as for the original weight matrix (Equation 1), but does not transform this frequency into a log-odd score. Instead the following formula was applied to transform the frequency matrix into a weight matrix:
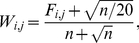
(2)where 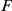 is the frequency matrix calculated by Predikin and 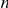 is the number of sequences used to calculate the frequency. It was originally believed that the standard Predikin weight matrix would contain additional information over the new style weight matrix.

### Frobenius Distance and p-Values

To assess the quality of Predikin's position weight matrices we used the same evaluation method as the DREAM4 challenge: similarity to a experimentally mapped position weight matrix using the distance induced by the Frobenius norm. The Frobenius norm is equal to the square root of the matrix trace of 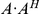, where 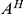 is the conjugate transpose of 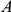, that is, 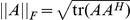 where 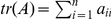. Effectively, the predicted position weight matrix is subtracted from the “gold standard” (experimentally derived) and the Frobenius norm for the resulting matrix is calculated. The DREAM4 challenge also provided p-values for each Frobenius distance, this is the probability that a random position weight matrix has the same or smaller Frobenius distance.

### Availability

Predikin is available as a web-server at http://predikin.biosci.uq.edu.au.
